# Massive expansion and diversity of nicotinic acetylcholine receptors in lophotrochozoans

**DOI:** 10.1186/s12864-019-6278-9

**Published:** 2019-12-05

**Authors:** Yu Jiao, Yanfei Cao, Zhe Zheng, Ming Liu, Ximing Guo

**Affiliations:** 1 Fishery College, Guangdong Ocean University, Zhanjiang, 524025 Guangdong China; 2 Haskin Shellfish Research Laboratory, Department of Marine and Coastal Sciences, Rutgers University, 6959 Miller Avenue, Port Norris, NJ 08349 USA

**Keywords:** Nicotinic acetylcholine receptors, Cholinergic signaling, Gene expansion, Retroposition, Tandem duplication, Adaptation, Oyster, Bivalve, Mollusca

## Abstract

**Background:**

Nicotinic acetylcholine receptors (nAChRs) are among the oldest and most conserved transmembrane receptors involved in signal transduction. Despite the prevalence and significance of cholinergic signaling, the diversity and evolution of nAChRs are not fully understood.

**Result:**

By comparative genomic analysis, we found massive expansions of nAChR genes in molluscs and some other lophotrochozoans. The expansion is particularly pronounced in stationary bivalve molluscs with simple nervous systems, with the number of nAChR genes ranging from 99 to 217 in five bivalves, compared with 10 to 29 in five ecdysozoans and vertebrates. The expanded molluscan nAChR genes tend to be intronless and in tandem arrays due to retroposition followed by tandem duplication. Phylogenetic analysis revealed diverse nAChR families in the common ancestor of bilaterians, which subsequently experienced lineage-specific expansions or contractions. The expanded molluscan nAChR genes are highly diverse in sequence, domain structure, temporal and spatial expression profiles, implying diversified functions. Some molluscan nAChR genes are expressed in early development before the development of the nervous system, while others are involved in immune and stress responses.

**Conclusion:**

The massive expansion and diversification of nAChR genes in bivalve molluscs may be a compensation for reduced nervous systems as part of adaptation to stationary life under dynamic environments, while in vertebrates a subset of specialized nAChRs are retained to work with advanced nervous systems. The unprecedented diversity identified in molluscs broadens our view on the evolution and function of nAChRs that are critical to animal physiology and human health.

## Background

Nicotinic acetylcholine (ACh) receptors (nAChRs) are members of a superfamily of pentameric ligand-gated ion channel proteins that include gamma aminobutyric acid receptors, glycine receptors, 5-hydroxytryptamine receptors, and some invertebrate glutamate receptors. Characterized by the conserved cystine bridge separated by 13 amino acid residues, this large group of proteins is also referred to as the “Cys-loop receptor superfamily” [1, 2]. nAChRs respond to the neurotransmitter ACh as well as nicotine, differing from muscarinic acetylcholine receptors (mAChRs). Since their first discovery in *Torpedo californica* and *Torpedo marmorata* in the early 1980s [3], nAChRs have been identified in all vertebrates and invertebrates studied. nAChR proteins consist of five subunits. In humans, 17 nAChR subunit genes have been identified [4, 5]. The fruit fly *Drosophila melanogaster* has 10 nAChR subunit genes [6]. In molluscs, 12 nAChR subunit genes have been reported in the snail *Lymnaea stagnalis* [7], and two have been identified in the bivalve *Chlamys farreri* [8]. nAChR proteins were also found in bacteria and plants [9]. All nAChR subunits possesses an N-terminal extracellular domain with the Cys-loop and other conserved sites for ligand binding, and four transmembrane regions (M1–4) responsible for ion channel, receptor localization and modulation of receptor function. The five subunits are symmetrically arranged around a central ion channel [10], which mediates the flux of cations Na^+^, K^+^, and Ca^2+^ when stimulated endogenously by ACh [11].

In mammals, nAChRs are widely distributed in the nervous system, where they regulate neurotransmitter release, cell excitability and neuronal integration, which are crucial for network operations and physiological homeostasis related to anxiety, sleep, food intake, fatigue, the processing of pain, immune and stress responses, and a number of cognitive functions such as memory, selective attention and emotional processing [12–14]. Decline, disruption, or alterations of nicotinic cholinergic regulation network may lead to various diseases such as epilepsy, Parkinson’s disease, Alzheimer’s disease, inflammation and addiction [15–17]. nAChRs are also found in non-nervous systems, such as muscle, macrophages, lymphoid tissue and skin [14]. An important role for alpha-7 nAChR is modulating inflammatory response, where disruption of its expression in vivo significantly increases the release of endotoxin-induced tumor-necrosis factor in humans [18]. In the bivalve mollusc *C. farreri*, two nAChR genes were detected in all organs including adductor muscle, mantle, gill, hepatopancreas, kidney and gonad, and their expression increased after LPS and TNF-a stimulation, indicating a role in immunomodulation [8]. In both oysters and scallops, ACh and AChRs may regulate immune response through the neuroendocrine-immune system [19–21]. In insects, nAChRs are expressed throughout the central nervous system and play crucial roles in escape behaviors, learning, memory and olfactory [6, 22, 23]. In bacteria, nAChR homologous ligand-gated ion channels were reported as proton-gated ion channels and might contribute to adaptation to pH change [24]. ACh and its receptors belong to one of the oldest signaling pathway, regulating basic cellular functions such as proliferation, differentiation and cytoskeletal organization [9, 14, 25].

Mollusca is the second largest phylum of Animalia, accounting for about 23% of all the named marine animal species [26]. Molluscs are widely distributed in diverse marine, freshwater and terrestrial environments. Their remarkable adaptation to highly variable or stressful environments is not well understood at molecular and genomic levels [27]. Most molluscs, with the exception of cephalopods, have a relatively simple nervous system, probably in adaptation to stationary benthic or epibenthic life. While nAChRs are a crucial component of the most important and phylogenetically conserved cholinergic system, their roles in molluscan biology and adaptation are largely unknown. The involvement of nAChRs in bivalve immune response has been suggested but not well studied. Studies of nAChRs in molluscs may help us to understand how molluscs respond and adapt to diverse environments with simple nervous systems. The genomes of several molluscs, such as *Crassostrea gigas* [28], *Pinctada fucata martensii* [29], *Mizuhopecten yessoensis* [30], *Modiolus philippinarum* and *Bathymodiolus platifrons* [31], have recently been sequenced, providing an opportunity to study the diversity and function of molluscan nAChRs. Our analysis of available genomic and transcriptomic data revealed a massive expansion and diversification of nAChR genes in molluscs and some other lophotrochozoans, possibly in adaptation to stationary life under variable environments.

## Results

### Massive expansion of nAChRs in molluscs

**Fig. 1:**
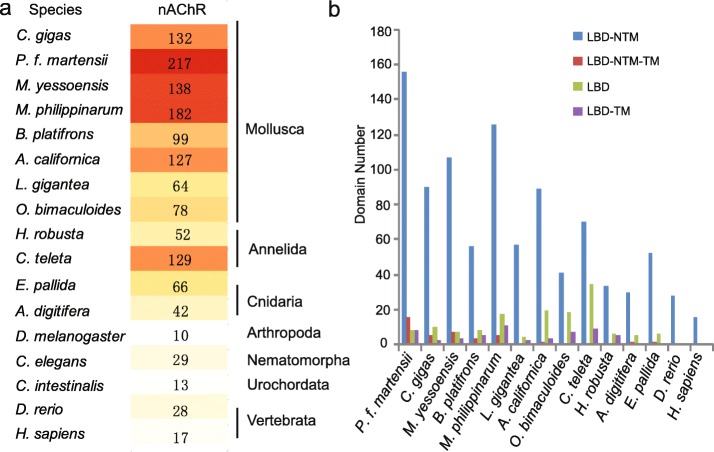
Expansion of nAChR genes in molluscs. **a** Number of nAChR genes in different species. **b** Common domain combinations of nAChRs in different species. LBD: neurotransmitter-gated ion-channel ligand binding domain; NTM: neurotransmitter-gated ion-channel transmembrane region; TM: transmembrane domain

Homology-based annotation with InterProScan, KEGG, Nr and manual corrections identified a surprisingly large number of nAChR genes in various species. Compared with model species from Ecdysozoa and Deuterostomia, a massive expansion of nAChR genes was found in molluscs (Fig. 1a). The expansion was particularly pronounced in stationary bivalve molluscs with simple nervous systems, with the number of nAChR genes ranging from 99 to 217 in five bivalves, compared with 10 to 29 in five ecdysozoans and vertebrates. A significant expansion of nAChR genes was also observed in Annelida (52–129), another branch of Lophotrochozoa.

Conserved domain analysis with Simple Modular Architecture Research Tool (SMART) revealed that the typical neurotransmitter-gated ion-channel ligand binding domain (LBD) and neurotransmitter-gated ion-channel transmembrane domains (NTM) structure was conserved in the majority (69.4%) of nAChRs from all species (Fig. 1b, Table 1). The expanded nAChRs from molluscs are highly diverse in domain structure. While all nAChRs from *Danio rerio* and *Homo sapiens* had the typical LBD-NTM domain structure, 33 different domain combinations were observed in the expanded nAChRs of molluscs, annelids and cnidarians (Table 1). Deviations in domain structures mostly involved the loss or duplication of the LBD or NTM domains, or the presence of a different transmembrane (TM) domain. Some molluscan nAChR genes, 8 in *C. gigas* and 9 in *P. f. martensii*, contained other functional domains, such as dynamin and GPCR-autoproteolysis inducing (GAIN) domains, which are not found in nAChRs of other species and may support novel functions.

**Table 1:** Domain combination in nAChR genes from different species

Domain combinations	*P. f. martensii*	*C. gigas*	*M. yessoensis*	*B. platifrons*	*M. philippinarum*	*L. gigantea*	*A. californica*	*O. bimaculoides*	*C. teleta*	*H. robusta*	*A. digitifera*	*E. pallida*	*D. rerio*	*H. sapiens*
LBD-NTM	156	90	107	56	126	57	89	41	70	33	30	52	28	16
LBD-NTM-TM	16	5	7	3	5	0	1	0	0	0	1	1	0	0
LBD	8	10	7	8	17	4	19	18	34	6	5	6	0	0
LBD-TM	8	2	3	5	11	2	3	7	9	5	0	0	0	0
NTM	3	0	0	6	5	0	4	6	1	2	1	0	0	0
NTM-TM	4	4	1	0	1	0	4	0	0	0	2	3	0	0
TM-LBD-NTM	1	1	4	0	1	0	1	0	1	2	0	1	0	0
TM-LBD-NTM-TM	4	1	4	1	2	0	1	0	0	0	0	0	0	0
TM-LBD	1	0	0	1	0	0	0	1	2	0	0	0	0	0
LBD-NTM-LBD-NTM-LBD-NTM	1	0	0	0	0	0	0	0	0	0	0	0	0	0
TM-NTM	0	0	0	0	0	0	0	1	1	0	0	0	0	0
LBD-LBD-NTM	1	2	1	1	1	0	0	0	2	0	0	2	0	0
LBD-NTM-LBD	1	0	0	1	1	0	0	0	0	0	0	0	0	0
LBD-LBD-NTM-TM	2	1	0	1	1	0	0	0	2	0	0	0	0	0
LBD-NTM-LBD-MTM-TM	1	2	0	0	0	0	0	0	0	0	0	0	0	0
LBD-TM-LBD-NTM	1	0	0	0	0	0	0	0	0	0	0	0	0	0
LBD-NTM-LBD-NTM-LBD-NTM-TM	0	1	0	0	0	0	0	0	0	0	0	0	0	0
LBD-NTM-LBD-NTM	0	1	1	2	0	0	0	0	0	0	1	0	0	0
LBD-NTM-NTM	0	1	0	0	0	0	0	1	4	0	0	0	0	0
LBD-NTM-TM-LBD-NTM-TM	0	2	0	1	1	0	0	0	0	0	0	0	0	0
LBD-TM-LBD-TM	0	1	0	0	0	0	0	0	0	0	0	0	0	0
TM-LBD-TM	0	0	0	2	0	0	0	1	1	2	0	0	0	0
LBD-LBD-TM	0	0	0	1	0	0	0	0	0	0	0	0	0	0
LBD-LBD	0	0	0	1	0	0	0	0	0	0	0	0	0	0
LBD-NTM-NTM-TM	0	0	0	1	0	0	1	0	0	0	0	0	0	0
NTM-TM-LBD-NTM-TM	0	0	0	1	0	0	0	0	0	0	0	0	0	0
LBD-TM-LBD-NTM-TM	0	0	0	1	0	0	0	0	0	0	0	0	0	0
LBD-TM-NTM	0	0	0	0	1	0	1	0	1	0	0	0	0	0
TM-NTM-TM	0	0	0	0	1	0	1	0	0	0	0	0	0	0
LBD-NTM-TM-LBD-NTM	0	0	0	0	1	0	0	0	0	0	0	0	0	0
LBD-NTM-LBD-NTM-TM	0	0	0	0	1	0	0	0	0	0	0	0	0	0
TM-LBD-TM-LBD-NTM-TM	0	0	0	0	1	0	0	0	0	0	0	0	0	0
LBD-LBD-TM-LBD-NTM	0	0	0	0	0	0	0	0	1	0	0	0	0	0
LBD-LBD-NTM-NTM	0	0	0	0	0	0	0	0	0	0	0	0	0	1
with other functional domains	9	8	3	6	5	1	2	2	0	2	2	1	0	0
	217	132	138	99	182	64	127	78	129	52	42	66	28	17

### Evolution of nAChR gene families

**Fig. 2:**
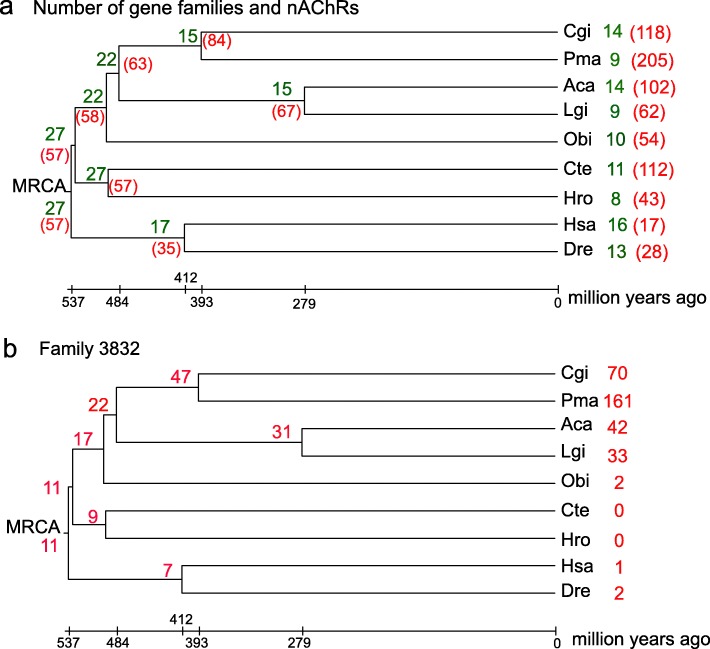
Evolution of nAChR gene families. **a** Phylogenetic tree of 644 nAChR genes with the typical LBD-NTM domain structure (without other functional domains) from nine species. The number of nAChR families is in *green*, and the number of nAChR genes is in *red*. MRCA represents the most recent common ancestor. **b** Phylogenetic tree of Family 3832 with the number of nAChR genes in *red*. Cgi: *C. gigas*, Pma: *P. f. martensii*, Lgi: *L. gigantea*, Aca: *A. californica*, Obi: *O. bimaculoides*, Hro: *H. robusta*, Cte: *C. teleta*, Dre: *D. rerio* and Hsa: *H. sapiens*

Gene family analysis was conducted with deduced amino acid sequences of all protein coding genes from nine species: *C. gigas*, *P. f. martensii*, *Lottia gigantea*, *Aplysia californica*, *Octopus bimaculoides*, *Helobdella robusta*, *Capitella teleta*, *D. rerio* and *H. sapiens*. All genes were grouped into 36,841 families. This analysis identified 57 nAChR orthologs grouped into 27 families present in the most recent common ancestor (MRCA) of bilaterians (Fig. 2a). During evolution, the number of gene families decreased in all lineages, while the number of nAChR genes increased in molluscs and annelids. In humans, the number of gene families reduced to 16 and the number of genes reduced to 17. In molluscs, the number of gene families decreased to 9–14, but the number of typical nAChR genes expanded to 205 in *P. f. martensii*, 118 in *C. gigas* and 102 in *A. californica*.

Most of the expansion and contraction of nAChR genes are lineage-specific as exemplified by Family 3832. This family has 316 nAChR genes in the nine extant species analyzed, which can be traced back to 11 orthologs in the MRCA of bilaterians (Fig. 2b). The number of genes in Family 3832 expanded to 17 in the MRCA of Mollusca, 47 in the MRCA of Bivalvia, 70 in *C. gigas*, and 161 in *P. f. martensii.* The family also expanded in Gastropoda albeit to a lesser extent. Family 3832 contracted in *O. bimaculoides, H. sapiens* and *D. rerio*, and completely lost in two ecdysozoans, *Caenorhabditis elegans* and *D. melanogaster*.

### Massive intronless nAChR genes in molluscs

**Fig. 3:**
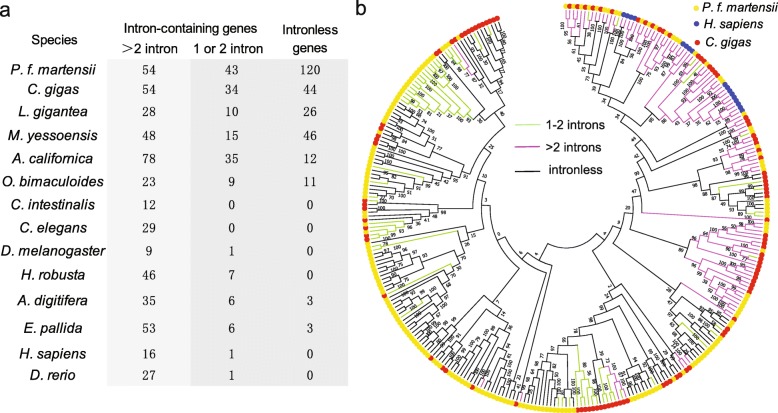
Massive intronless nAChR genes in molluscs. **a** Number of nAChR genes with > 2, 1–2 and no introns in different species. **b** Neighbor-joining tree of nAChRs from *C. gigas, P. f. martensii* and *H. sapiens,* showing lineage-specific expansion and close relationship between nAChR genes with no or 1–2 introns

Exon-intron structure analysis revealed large numbers of intronless nAChR genes in molluscs. The number of intronless nAChR genes in molluscs ranged from 11 to 120, while intronless nAChRs were not observed in *H. sapiens, D. rerio, C. intestinalis, C. elegans, D. melanogaster* and *H. robusta* (Fig. 3a). Further, many nAChR genes from molluscs (9–43) had only one or two introns, compared to an average of 6.8 introns per gene in humans (Additional file 1: Figure S1). In *C. gigas*, 44 (33.3%) of the 132 nAChRs were intronless, and 34 (25.8%) nAChRs contained only 1–2 introns. In *P. f. martensii*, 120 (55.3%) of the 217 nAChRs were intronless, and 43 nAChRs (19.8%) had only 1–2 introns (Fig. 3a). The intronless genes are likely retrogenes from retroposition, and genes with 1–2 introns may be retrogenes that retained or gained 1–2 introns.

**Fig. 4:**
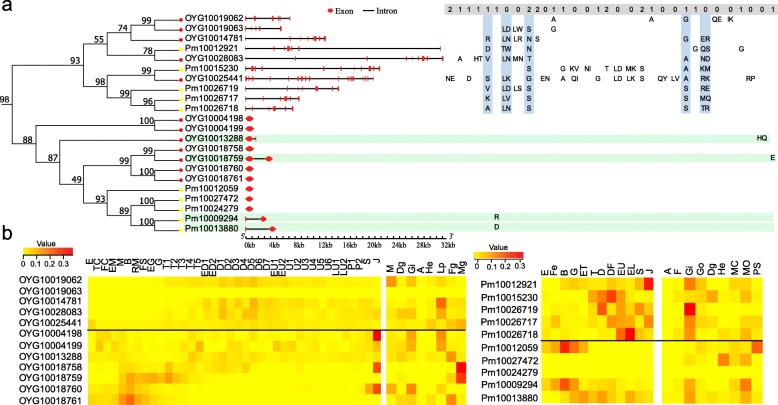
Intron position and expression of one cluster of nAChR genes. **a** Phylogenetic relationship, the corresponding gene structure and intron position of one group of nAChRs, containing both of intron-rich (> 2 introns) and intron-poor nAChRs (≤2 introns). The intron positions shaded *blue* are conserved. Genes shaded in *green* have only one intron. Numbers (0, 1, 2) above the intron position indicate intron insertion phases: 0, between two consecutive codons; 1, between the first and second codon position, and 2, between the second and third codon position. **b** Heat maps of expression at different developmental stages and in different organs in *C. gigas* (*left*) and *P. f. martensii* (*right*). Genes above the *black line* are intron-rich, and genes below the *black line* are intron-poor nAChRs. Scale bars refer to relative expression. E, egg; TC, two cells; FC, four cells; EM, early morula; M, morula; B, blastula; RM, rotary movement; FS, free swimming; EG, early gastrula stage; G, gastrula; T1, trochophore 1; T2, trochophore 2; T3, trochophore 3; T4, trochophore 4; T5, trochophore 5; ED1, early D-larva 1; ED2, early D-larva 2; D1, D-larva 1; D2, D-larva 2; D3, D-larva 3; D4, D-larva 4; D5, D-larva 5; D6, D-larva 6; D7, D-larva 7; EU1, early umbo larva 1; EU2, early umbo larva 2; U1, umbo larva 1; U2, umbo larva 2; U3, umbo larva 3; U4, umbo larva 4; U5, umbo larva 5; U6, umbo larva 6; LU1, late umbo larva 1; LU2, late umbo larva 2; P1, pediveliger 1; P2, pediveliger 2; S, spat; J, juvenile; Fe, fertilization; B, blastula; G, gastrula; ET, early trochophore; DF, D-larvae before feeding; EU, early umbo larvae; PV, post-veliger; CT, 2–8 cell stage; M, mantle; Di, digestive gland; A, adductor muscle; F, foot; Go, gonad; Fg, female gonad; Mg, male gonad; PS, pearl sac at 180 d; MP, mantle pallium; MC, mantle central; He, hemocyte; Lp, labial palp

Phylogenetic analysis of 307 nAChR genes from *C. gigas*, *P. f. martensii* and *H. sapiens* also showed that the expansion was mostly lineage-specific (Fig. 3b). Paralogs within the same species were mostly clustered together, indicating that their expansions occurred after the speciation event. This finding is consistent with results from gene family analysis (Fig. 2). Furthermore, intron-rich nAChR genes (> 2 introns) were clustered together, and intronless nAChRs were clustered together and with nAChRs with 1–2 introns, suggesting that the latter two had the same origin. Detailed analysis of one cluster showed that some of the intron positions are conserved in the intron-rich nAChRs, while intron positions in the one-intron only nAChRs were novel, probably representing newly gained introns after retroposition (Fig. 4a). These findings support an evolutionary path where intronless nAChRs are derived from intron-rich nAChRs by retroposition, nAChRs with 1–2 introns are derived from intronless nAChRs through intron gains, and intron-rich nAChRs may also experience some intron gain or loss during evolution.

Within the same cluster, intron-rich and intron-poor nAChR genes differed in temporal and spatial expression profiles, indicative of divergence in regulatory elements and possibly function. In both *C. gigas* and *P. f. martensii*, some intron-poor nAChRs expressed during embryonic development before trochophore stage and the development of the nervous system, while intron-rich nAChRs expressed at D- and late larval stages (Fig. 4b). In *C. gigas,* three intron-poor nAChRs were highly expressed in juveniles and two in male gonad. In *P. f. martensii*, two intron-poor nAChRs were highly expressed at early embryonic stages, and several intron-rich nAChRs were highly expressed in late larvae, juveniles and adult gills (Fig. 4b). The difference in expression profile may indicate divergence in regulation or function. Overall, expression analysis of all nAChR genes from *C. gigas* and *P. f. martensii* at different development stages and in different organs showed that more intron-poor nAChRs had no or low expression (< 1 RPKM, Reads Per Kilobase per Million mapped reads) than intron-rich nAChRs, 7.7% vs. 3.6 and 31% vs. 7.5% in *C. gigas* and *P. f. martensii*, respectively, which is consistent with inactive pseudogenes from retroposition.

### Tandem duplication of nAChR genes

Of the 132 nAChR genes in *C. gigas*, 72 (55%) are linked in tandem arrays including 16 two-gene pairs, five three-gene arrays, and one array each for four-, six-, seven- and eight-gene arrays (Table 2). In *P. f. martensii*, 140 (65%) of the 217 nAChR genes are present in tandem arrays: 18 two-gene pairs, 11 three-gene arrays, 2–3 arrays of 4–6 genes, one array of seven genes and two arrays of 12 genes. Among the tandemly arrayed nAChRs, 49 (68.1%) in *C. gigas* and 115 (82.1%) in *P. f. martensii* are intron-poor nAChRs. Among the single-copy nAChRs, 29 (48.3%) and 48 (62.3%) are intron-poor nAChRs in *C. gigas* and *P. f. martensii*, respectively. Thus, in addition to retroposition, tandem duplication is also a major contributor to the massive expansion of nAChR genes in molluscs.

**Table 2:** Tandem duplication of nAChR genes in *C. gigas* and *P. f. martensii*

Species	Type	No. of Arrays	No. of Genes
*C. gigas*	2-gene array	16	32
	3-gene array	5	15
	4-gene array	1	4
	6-gene array	1	6
	7-gene array	1	7
	8-gene array	1	8
	total	25	72 (54.5%)
*P. f. martensii*	2-gene array	18	36
	3-gene array	11	33
	4-gene array	3	12
	5-gene array	2	10
	6-gene array	3	18
	7-gene array	1	7
	12-gene array	2	24
	total	40	140 (65.0%)

**Fig. 5:**
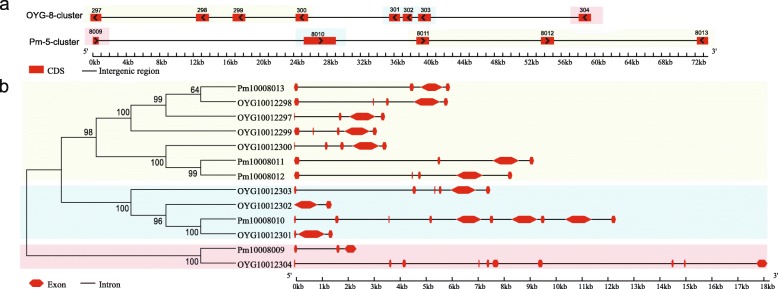
Stepwise and lineage-specific tandem duplication of nAChR genes in *C. gigas* and *P. f. martensii*. **a** Arrangement of nAChR genes in two tandemly duplicated arrays from *C. gigas* and *P. f. martensii*. *Arrows* represent gene direction in the scaffolds. Numbers above the *red box* are gene IDs of nAChRs, for example, 297 is OYG10012297. **b** Phylogenetic relationship and corresponding gene structure of tandemly duplicated nAChR genes. OYG: *C. gigas*; Pm: *P. f. martensii*

Analysis of two tandem arrays of duplicated nAChRs in *C. gigas* and *P. f. martensii* indicated that the tandem duplication was stepwise and lineage-specific. The two arrays originated in the common ancestor of the two bivalves as indicated by sequence homology and similarities in gene structure (Fig. 5a, b). The ancestral array possibly had six tandemly duplicated nAChRs and after the divergence of the two species, paralogs Pm1008011 and Pm10008012 emerged by tandem duplication, and orthologs of OYG10012297 and OYG10012299 were lost in *P. f. martensii*. OYG10012301, OYG10012302 and OYG10012303 are fragments of the same gene, which is orthologous to Pm10008010. While the number of genes in the ancestral array is uncertain, the synteny and correspondence between sequence homology and position in the arrays support stepwise tandem duplication. OYG10012304 is most homologous with a7nAChR, the most ancient nAChR [32, 33], but the corresponding gene in *P. f. martensii*, Pm10008009, has degenerated with only NTM domain remaining.

### Sequence diversity of nAChRs

The massive expansion has resulted in high sequence diversity of the expanded nAChR genes. In *C. gigas*, protein length of nAChRs varied greatly from 66 to 2013 aa, compared with 458 to 627 aa in humans [34]. Among the 141 LBD domains found in *C. gigas*, eight did not have the Cys-loop, which was critical for the function of the ligand-gated ion channel. The sequences of the Cys-loop from *C. gigas* are more diverse than that from humans (Additional file 2: Figure S2). In human, 10 of the 17 nAChRs contain the two characteristic cysteine residues and are recognized as alpha nAChRs, and 8 alpha nAChRs contain the conserved principal binding sites for ACh (Additional file 3: Figure S3). In *C. gigas*, 33 of the 132 nAChRs are alpha nAChRs, while only 13 of the 33 have the principal binding sites completely conserved (Additional file 4: Figure S4). The high sequence diversity in the LBD may support the binding to diverse ligands for signal transduction.

### Functional diversity of nAChRs in molluscs

**Fig. 6:**
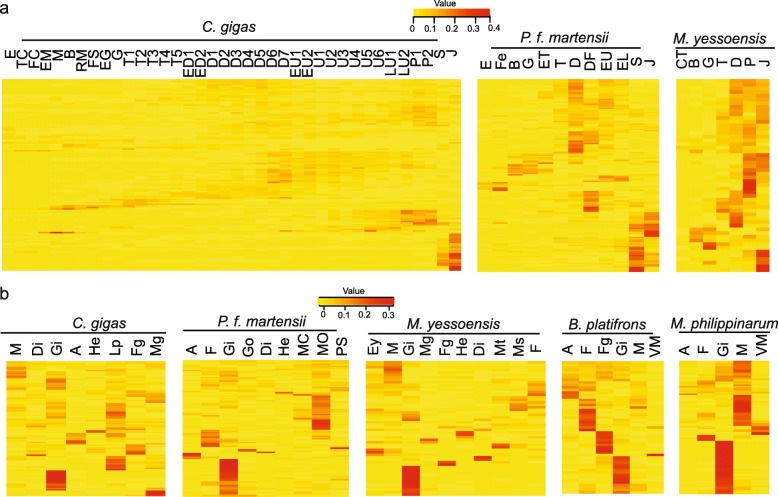
Diversity in temporal and spatial expression of nAChR genes in molluscs. **a** Heat maps nAChR expression during development in *C. gigas*, *P. f. martensii* and *M. yessoensis*. **b** expression patterns of nAChR genes in different tissues in *C. gigas*, *P. f. martensii*, *M. yessoensis*, *B. platifrons* and *M. philippinarum*. E, egg; TC, two cells; FC, four cells; EM, early morula; M, morula; B, blastula; RM, rotary movement; FS, free swimming; EG, early gastrula; G, gastrula; T1, trochophore 1; T2, trochophore 2; T3, trochophore 3; T4, trochophore 4; T5, trochophore 5; ED1, early D-larva 1; ED2, early D-larva 2; D1, D-larva 1; D2, D-larva 2; D3, D-larva 3; D4, D-larva 4; D5, D-larva 5; D6, D-larva 6; D7, D-larva 7; EU1, Early umbo larva 1; EU2, Early umbo larva 2; U1, umbo larva 1; U2, umbo larva 2; U3, umbo larva 3; U4, umbo larva 4; U5, umbo larva 5; U6, umbo larva 6; LU1, late umbo larva 1; LU2, late umbo larva 2; P1, pediveliger 1; P2, pediveliger 2; S, spat; J, juvenile; Fe, fertilization; ET, early trochophore; DF, D-larvae before feeding; EU, early umbo larvae; PV, post-veliger; CT, 2–8 cells. M, mantle; Di, digestive gland; A, adductor muscle; F, foot; Go, gonad; Fg, female gonad; Mg, male gonad; PS, pearl sac at 180 d; MP, mantle pallium; MC, mantle central; He, hemocyte; Lp, labial palp; Ey, eye; Ms., smooth muscle; Mt, striated muscle; VM, visceral mass

By responding to endogenous ACh, nAChRs regulate a wide range of biological processes and influence a number of physiological functions. Analysis of the developmental transcriptomes of *P. f. martensii* indicated that 4 nAChR genes were highly expressed in fertilized eggs, 32 nAChRs were highly expressed at D-stage, along with some nAChRs highly and specifically expressed at other developmental stages (Fig. 6a, Additional file 7: Table S1). In *C. gigas*, 10 nAChRs were highly expressed before or during the trochophore stage and before the development of the nervous system, and 21 nAChRs were only expressed after spat stage. Development transcriptomes of scallop also showed that some nAChRs were expressed in a stage-specific manner (Fig. 6a, Additional file 7: Table S1).

Analysis of organ transcriptomes revealed that the expression of many nAChR genes are organ-specific, which may reflect diversification in spatial regulation or functional compartmentalization. In *C. gigas*, 20 nAChRs were highly expressed in the gill, 3 nAChRs were only expressed in the male gonad, and 7 nAChRs showed high expression in the female gonad (Fig. 6b, Additional file 7: Table S1). In all bivalve molluscs studied, gills showed the highest expression of nAChRs compared with other organs (Fig. 6b, Additional file 7: Table S1). In gills of *C. gigas*, the expression of many nAChRs responded to changes in environmental conditions such as water temperature, salinity and air exposure (Additional file 5: Figure S5). Some nAChRs were up-regulated in response to infections by *Vibrio* species (*V. anguillarum*, *V. tubiashii*, *V. aestuarianus*, *V. alginolyticus*) and Ostreid herpesvirus 1-μVar, suggesting that they play a role in immune response (Additional file 6: Figure S6).

### Synthesis of the ACh in organs and during development

**Fig. 7:**
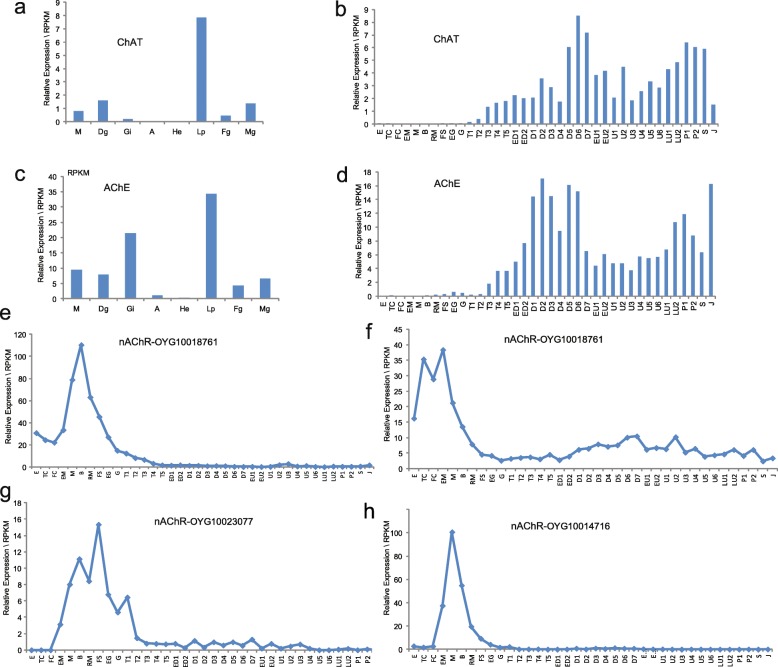
Expression of ChAT, AChE and four nAChR genes in different organs and during early development in *C. gigas*. **a** and **b** Expression of ChAT (choline O-acetyltransferase) in different organs and during early development. **c** and **d** Expression of AChE (actylcholinesterase) in different organs and during early development. **e**–**h** High and specific expression of four nAChR genes before trochophore stage. E, egg; TC, two cells; FC, four cells; EM, early morula; M, morula; B, blastula; RM, rotary movement; FS, free swimming; EG, early gastrula; G, gastrula; T1, trochophore 1; T2, trochophore 2; T3, trochophore 3; T4, trochophore 4; T5, trochophore 5; ED1, early D-larva 1; ED2, early D-larva 2; D1, D-larva 1; D2, D-larva 2; D3, D-larva 3; D4, D-larva 4; D5, D-larva 5; D6, D-larva 6; D7, D-larva 7; EU1, early umbo larva 1; EU2, early umbo larva 2; U1, umbo larva 1; U2, umbo larva 2; U3, umbo larva 3; U4, umbo larva 4; U5, umbo larva 5; U6, umbo larva 6; LU1, late umbo larva 1; LU2, late umbo larva 2; P1, pediveliger 1; P2, pediveliger 2; S, spat; J, juvenile. M, mantle; Di, digestive gland; A, adductor muscle; F, foot; Go, gonad; Fg, female gonad; Mg, male gonad; Lp, labial palp

Choline O-acetyltransferase (ChAT), a transferase enzyme responsible for the synthesis of ACh, was not expressed in the gill but highly expressed in the labial palp (Fig. 7a). This finding suggests that labial palp, a hemolymph sinus located close to the cerebral ganglia, may play a major role in ACh synthesis and distribution in bivalves. Acetycholinesterase (AChE), a hydrolytic enzyme catalyzing the degradation of ACh into acetate and choline [35], was highly expressed in both labial palp and gill (Fig. 7c), as expected. The high expression of AChE and low expression of ChAT in the gill of *C. gigas* imply that ACh is produced in labial palp and transported to the gill where most of the nAChR genes are expressed.

During early development of *C. gigas*, ChAT and AChE were not expressed (Fig. 7b and d) before trochophore stage, when some nAChRs were highly expressed including four specifically expressed between 2-cell and blastula stages (Fig. 7e–h), raising the question what ligands these nAChRs recognize in the absence of endogenous ACh (under external fertilization) and the nervous system.

## Discussion

Our analysis of a comprehensive set of genomic and transcriptomic data revealed massive expansion and diversity of nAChR genes in molluscs as well as some annelids, compared with ecdysozoans and deuterostomes. Prior to this study, only 14 nAChR genes have been reported and characterized in molluscs [7, 8]. The finding of large numbers of nAChR genes in molluscs is surprising and provides an opportunity to study the evolution of this important class of receptors. The largest expansion of nAChR genes is found in bivalve molluscs with simple nervous systems, 138 in scallop *M. yessoensis*, 182 in mussel *M. philippinarum*, 132 in oyster *C. gigas* and 217 in pearl oyster *P. f. martensii*, while species with advanced nervous systems has small numbers of nAChRs: 78 in octopus *O. bimaculoides,* 10 in fruit fly *D. melanogaster*, 28 in zebrafish *D. rerio* and 17 in *H. sapiens.* This is intriguing because, as neurotransmitter receptors, nAChRs in vertebrates primarily function in neurosignal transduction working closely with the nervous system. The finding of massive nAChRs in molluscs without advanced nervous systems suggests that nAChRs may have broad functions and are critical to the adaptation of molluscs.

The cholinergic system with nAChRs at its core is one of the oldest signaling pathways that emerged long before the development of the nervous system [9]. nAChRs originated from the pentameric ligand-gated ion channels (pLGICs) of prokaryotes [24]. In the cyanobacterium *Gloebactor violaceus*, pLGIC is involved in proton transport and adaptation to pH changes. A phylogenetic analysis of nAChRs from vertebrates and ecdysozoans revealed two gene duplication events before the divergence of the two lineages [36]. Our analysis of a broad data set including molluscs, annelids, cnidarian as well as ecdysozoans and vertebrates identified 27 families of nAChRs in the MRCA of bilaterians, more than that in any extent group. This finding indicates that the MRCA of bilaterians and possibly metazoans, which probably had a primitive nervous system, had a large and highly diverse repertoire of nAChRs. Many of these ancient nAChRs might have had broad functions outside the nervous system as the nervous system was not well developed. During subsequent evolution and divergence, the number of gene families declined in all lineages, and the number of nAChR genes greatly expanded in bivalve molluscs but reduced in ecdysozoans and deuterostomes. Therefore, we hypothesize that the common ancestor of bilaterian had a large and diverse repertoire of nAChRs, probably with broad functions in development, immune and environmental responses, only a subset of specialized nAChRs were retained in ecdysozoans and vertebrates in association with advanced nervous systems, while some families greatly expanded in bivalve molluscs in adaptation to stationary life under variable environments.

Stationary or sessile organisms such as bivalve molluscs cannot use avoidance and have to reply on physiological adjustments for coping with adverse conditions. Bivalve molluscs have no adaptive immunity but thrive in microbe-rich environments as filter-feeders. Thus, bivalve molluscs such as oysters have developed remarkable tolerance to biotic and abiotic stresses [27]. The adaptation and resilience of bivalve molluscs are supported by the expansion and diversity of many stress and immune related genes [27, 28, 37, 38]. The massive expansion and high diversity of nAChRs may give bivalve molluscs enhanced capacity to handle and respond to environmental stimuli. The expansion of nAChRs in molluscs appears to be associated with reduced mobility and increased environmental heterogeneity. Cephalopods and gastropods with more advanced nervous systems and enhanced mobility have fewer nAChR genes than stationary bivalves. Among bivalves, mussel *M. philippinarum* that lives in variable intertidal waters has 182 nAChR genes, while mussel *B. platifrons* that lives in a more stable deep-sea environment has 99 nAChR genes. Thus, the massive expansion and diversification of nAChRs in bivalve molluscs could be an important part of the adaptation to stationary life under variable environments in compensation for simple nervous systems. For bivalve molluscs, gill is the organ with the highest exposure to surrounding water and serves as the main interface between the organism and the environment. Thus, the exceptionally high expression of nAChR genes in the gill may be an adaptation for rapid response to dynamic environmental conditions. In the Pacific oyster, some of the expanded nAChRs in oysters are also involved in stress responses, and one is upregulated by both bacterial and viral infections, while others show pathogen-specific upregulations, supporting their involvement in immune response. nAChRs is known as a modulator of inflammatory responses [18], and their role in immunomodulation has been previously demonstrated in bivalve molluscs [8, 19–21].

Our analysis indicates that both retroposition and tandem duplication have contributed to the massive expansion of nAChR genes in bivalve molluscs, as indicated by high proportion of intronless or intron-poor genes and tandem arrays. Intron processing may delay regulatory responses, and genes requiring rapid expression in response to environmental challenges contain few introns [39]. Intronless opsins in bivalve *C. farreri* are considered as adaptation for efficient transcription in support for scallop’s unusually advanced multi-eye visual system [40]. Intronless genes in eukaryotic genomes may come from ancestral prokaryotic genes or retroposition of intron-containing genes [41]. Intronless histone genes are originated from Prokaryota [42]. Intronless nAChR genes are only found in molluscs, not in ecdysozoans and vertebrates, and they are homologous to the intron containing nAChR genes from molluscs, indicating that these intronless nAChR are derived from intron-rich nAChRs by retroposition in molluscs and not from Prokaryota. Retrogenes produced by retroposition have been considered inconsequential, because of the lack of regulatory regions [43]. However, increasing data show that retrogenes may evolve rapidly, assume novel functions and contribute to gene diversity. It has been shown that retrogenes play a crucial role in the diversification of transcriptome and proteome, and may be responsible for species-specific adaptation [44, 45].

The massive expansion of nAChR genes in bivalve molluscs, through both retroposition and tandem duplication, has led to extensive diversity in sequence, domain structure and expression profile, which may support functional diversity and adaptation. Sequence and domain structure diversity are essential for functional diversification. In fact, nAChRs together with glycine receptors, serotonin receptors, and some invertebrate glutamate receptors are all members of the Cys-loop superfamily, with a7 nAChR most homologous to the ancestral gene [46]. Serotonin receptors in *C. elegans* and the invertebrate inhibitory glutamate receptors appear to arise through mutations in the LBD that changed ligand specificity [47]. Mutations in the ion channel domain NTM may change the ion specificity of nAChRs [47]. As such, high sequence diversity of the LBD may have broadened the ligand specificity of the expanded molluscan nAChRs.

It should be noted that, beside nAChRs, mAChRs are another class of ACh receptors for cholinergic signaling. mAChRs are members of the G protein-coupled receptors (GPCRs), which are widely distributed in multiple organs and critical to the maintenance of central and peripheral cholinergic neurotransmission [48]. Only 3 and 4 mAChRs are found in *C. gigas* and *P. f. martensii*, respectively (data not shown), while large numbers of nAChRs are observed. Both of nAChR and mAChR regulate a wide variety of physiological responses, such as cellular proliferation, neuronal differentiation, and a number of cognitive functions [48]. mAChRs are characterized by their interaction with muscarine and use of an intracellular secondary messenger system to transmit signals inside cells. nAChRs are ligand-gated ion channels characterized by their interaction with nicotine and mediate fast signal transmission through a rapid increase in the membrane permeability to Na^+^, K^+^ and Ca^2+^, which in turn regulates diverse cellular processes [32]. The expansion of nAChRs rather than mAChRs suggests that fast acting nAChRs may be more important for the adaptation of molluscs. The massively expanded nAChRs could help bivalve molluscs with limited nervous systems to continuously monitor and rapidly respond to environmental challenges with a fast flux of ions that are particularly abundant in seawater.

## Conclusion

In conclusion, massive expansion and diversity of nAChR genes are observed in molluscs and annelids especially in stationary bivalve molluscs with simple nervous systems. The expansion through both retroposition and tandem duplication have created remarkable diversity in sequence, domain structure and expression profile, which may support diversified functions. Some of the expanded nAChR genes are expressed during early development, while others are involved in immune and stress responses. The particularly extensive expansion and diversity in bivalve molluscs may be an adaptation to stationary life under variable environment, in compensation for reduced nervous systems. Cholinergic receptors are critically important for neurosignal transmission that regulates diverse physiological processes. Mutations in nAChRs are implicated in important human diseases such as epilepsy, Parkinson’s disease, Alzheimer’s disease, cognitive disorders, addiction and inflammation [15–17]. This study reveals unprecedented diversity of nAChRs and provides insights into the evolution of the cholinergic signaling pathways. Further characterization of these diverse nAChRs may identify novel functions and improve our understanding of the cholinergic signaling pathway that is critical to animal physiology, immune response and human health.

## Methods

### Identification of nAChRs

Protein sequences of *L. gigantea*, *A. californica*, *O. bimaculoides*, *H. robusta*, *C. teleta*, *D. rerio* and *H. sapiens* were downloaded from NCBI database (https://www.ncbi.nlm.nih.gov/protein/). For annotation, we queried all proteins against the databases Nr and KEGG, using BLASTP at E-value <= 1-e5, and accepted results with the best scores for each query protein. InterProScan [49] was used to identify domain structure. The annotation of *P. f. martensii*, *C. gigas, M. philippinarum* and *B. platifrons* proteins was from the previous research [28, 29, 31]. nAChR from the above-mentioned 11 species were identified based on the annotation from InterProScan, Nr and KEGG databases. When the homology-based annotation with InterProScan, KEGG, Nr was inconsistent or inconclusive, sequences were analyzed or inspected manually to verify if the sequences contained the LBD or NTM domain. The nAChRs from *M. yessoensis*, *Exaiptasia pallida*, *Acropora digitifera*, *D. melanogaster*, *C. elegans* and *Ciona intestinalis* were extracted from the NCBI gene database (https://www.ncbi.nlm.nih.gov/gene/?term=gene) annotated as nAChR genes. Protein domains of all identified nAChRs were re-analyzed and confirmed with Simple Modular Architecture Research Tool (SMART) version 5.1 (http://smart.Embl-heidelberg.de/). Only genes homologous with nAChR (E-value <= 1-e5) and containing both LBD and NTM domains, or including at least one LBD or one NTM domain were considered as nAChRs.

### Gene family analysis

We conducted gene family analysis with gene sets for the following nine species: *C. gigas*, *P. f. martensii*, *L. gigantea*, *A. californica*, *O. bimaculoides*, *H. robusta*, *C. teleta*, *D. rerio* and *H. sapiens*. Gene families were identified based on Treefam using the following steps. First, all proteins from the nine species were combined for all-to-all alignment using BLASTP (E-value <= 1e-7), and Solar (inner) (−a prot2prot) was used to collect all matches into one group for each query sequence. If the identity between two genes is more than 30%, the two genes are considered as homologous [29]. The H-score was also used as another criterion to assess similarity. Genes are clustered into families using average distances for hierarchical clustering algorithm based on all-versus-all H-scores. A total of 644 nAChR genes that had the canonical LBD-NTM domain structure (without other functional domains) were used for nAChR gene family analysis.

### Phylogenetic analysis

Phylogenetic analysis of the above nine species were conducted using MrBayes [50]. Divergence time for the species tree was estimated with the MCMCTree program within PAML package [51], using the divergence time between *D. rerio* and *H. sapiens* based on fossil records as reference [52]. Gene family expansion and contraction among the nine species on the phylogenetic tree were determined with CAFE [53] against the latest common ancestor for each two branches.

For phylogenetic tree construction, nAChRs missing LBD and/or NTM domains and nAChRs having other functional domains, such as dynamin, were excluded. After filtering, 182 nAChR genes from *P. f. martensii*, 108 from *C. gigas*, and 17 from *H. sapiens* were used to construct the phylogenetic tree. Multiple sequence alignments were performed using ClustalW multiple alignment program. Phylogenetic trees were constructed using MEGA7.0 [54] (http://www.megasoftware.net) with the neighbor-joining (NJ) algorithm. Confidence values were obtained with bootstrapping with 1000 replications.

### Exon-intron structure analysis of nAChR genes

Structure information for *P. f. martensii* and *C. gigas* nAChR genes were obtained from published genome data [28, 29]. Structure information for nAChR genes of other species were downloaded from NCBI database. The exon and intron structure of nAChR genes were drew using the Gene Structure Display Server (GSDS, http://gsds.cbi.pku.edu.cn/) [53].

### Expression profile of nAChR genes

The transcriptome data from different organs, at different stages and under challenge by pathogens and different environmental conditions were collected from the published research [28–31, 37, 55] and analyzed to extract expression profile of nAChR genes. Heatmaps were generated using the R language package.

## Supplementary information

**Additional file 1: Figure S1.** Extron-intron structure of nAChR genes from *H. sapiens.*

**Additional file 2: Figure S2.** Multi-alignment of Cys-loop of nAChR genes from *C. gigas* and *H. sapiens.*

**Additional file 3: Figure S3.** Multi-alignment of alpha nAChR genes from *H. sapiens.*

**Additional file 4: Figure S4.** Sequence diversity at and around ACh binding sites in 33 nAChR genes of *C. gigas* with the sites conserved. Sequences marked by the *red underline* is the Cys-loop. Amino acids in *green boxes* are ACh binding sites. Hsa, *H. sapiens;* Tma, *Torpedo marmorata*. Genes in purple are nAChRs with completely conserved principal binding sites.

**Additional file 5: Figure S5.** Expression of six nAChR genes in gills of *C. gigas* under different environmental conditions (data from Zhang et al. 2012). **a** Seven days at 5–25 °C or 12 h at 30 and 35 °C; **b** Seven days under different salinities; and **c** Air exposure for different durations.

**Additional file 6: Figure S6.** Expression of nAChR genes in gills of *C. gigas* in response to infection by pathogens. Left, expression of 3 nAChRs at different times after *Vibrio* (*V. anguillarum*, *V. tubiashii*, *V. aestuarianus*, *V. alginolyticus*) challenge (data from Zhang et al. 2015); Right, expression of 3 nAChRs at different times after Ostreid herpesvirus 1-μVar challenge (data from He et al. 2015). Y-axes is expression relative to Time 0.

**Additional file 7: Table S1.** Expression of nAChR genes (RPKM > 1) in different organs and at different development stages in *C. gigas* and *P. f. martensii.*

## Data Availability

The genome and transcriptome data of *P. f. martensii* were downloaded from GigaDB (PRJNA283019). The genome data of *C. gigas* were downloaded from European Bioinformatics Institute (AFTI01000000), and the transcriptome data were downloaded from National Center for Biotechnology Information (NCBI) Gene Expression Omnibus (GSE31012). The genome data of *B. platifrons* and *M. philippinarum* were downloaded from NCBI (PRJNA328542 and PRJNA328544), and their transcriptomes data were downloaded from NCBI Short Read Archive (SRA) (SRP078287 and SRP078294, respectively). The transcriptome data of *M. yessoensis* were downloaded from NCBI SRA (SRX1026991, SRX2238787, SRX2250258 SRX2238809, SRX2250256, SRX2250257, SRX2250258, SRX2250259, SRX2251047, SRX2251049, SRX2251056, SRX2251057 and SRX2279546). The transcriptomes data of *C. gigas* after infection were downloaded from NCBI SRA (SRP019967). Protein sequences of *L. gigantea*, *A. californica*, *O. bimaculoides*, *H. robusta*, *C. teleta*, *D. rerio* and *H. sapiens* were downloaded from NCBI BioProject PRJNA259762, PRJNA209509, PRJNA305125, PRJNA259764, PRJNA175705, PRJNA13922 and PRJNA168, respectively.

## Abbreviations

A: adductor muscle
Aca: *A. californica*
ACh: acetylcholine
AChE: acetycholinesterase
B: blastula
Cgi: *C. gigas*
ChAT: choline o-acetyltransferase
CT: 2–8 cells
Cte: *C. teleta*
D1: D-larva 1
D2: D-larva 2
D3: D-larva 3
D4: D-larva 4
D5: D-larva 5
D6: D-larva 6
D7: D-larva 7
DF: D-larvae before feeding
Di: digestive gland
Dre: *D. rerio*
E: egg
ED1: early D-larva 1
ED2: early D-larva 2
EG: early gastrula
EM: early morula
ET: early trochophore
EU: early umbo larvae
EU1: early umbo larva 1
EU2: early umbo larva 2
Ey: eye
F: foot
FC: four cells
Fe: fertilization
Fg: female gonad
FS: free swimming
G: gastrula
GAIN: GPCR-autoproteolysis inducing
Go: gonad
He: hemocyte
Hro: *H. robusta*
Hsa: *H. sapiens*
J: juvenile
LBD: neurotransmitter-gated ion-channel ligand binding domain
Lgi: *L. gigantea*
Lp: labial palp
LU1: late umbo larva 1
LU2: late umbo larva 2
M: mantle
M: morula
mAChRs: muscarinic acetylcholine receptors
MC: mantle central
Mg: male gonad
MP: mantle pallium
MRCA: most recent common ancestor
Ms.: smooth muscle
Mt: striated muscle
nAChRs: nicotinic acetylcholine receptors
NTM: neurotransmitter-gated ion-channel transmembrane domains
Obi: *O. bimaculoides*
P1: pediveliger 1
P2: pediveliger 2
Pma: *P. f. martensii*
PS: pearl sac at 180 d
PV: post-veliger
RM: rotary movement
RPKM: Reads Per Kilobase per Million mapped reads
S: spat
SMART: Simple Modular Architecture Research Tool
T1: trochophore 1
T2: trochophore 2
T3: trochophore 3
T4: trochophore 4
T5: trochophore 5
TC: two cells
Tma: *Torpedo marmorata*
U1: umbo larva 1
U2: umbo larva 2
U3: umbo larva 3
U4: umbo larva 4
U5: umbo larva 5
U6: umbo larva 6
VM: visceral mass
