# Supplementary figures and images for: Massive expansion and diversity of nicotinic acetylcholine receptors in lophotrochozoans
Source: BMC Genomics. 2019 Dec 5;20:937. doi: 10.1186/s12864-019-6278-9 (PMC6896357; doi:10.1186/s12864-019-6278-9)

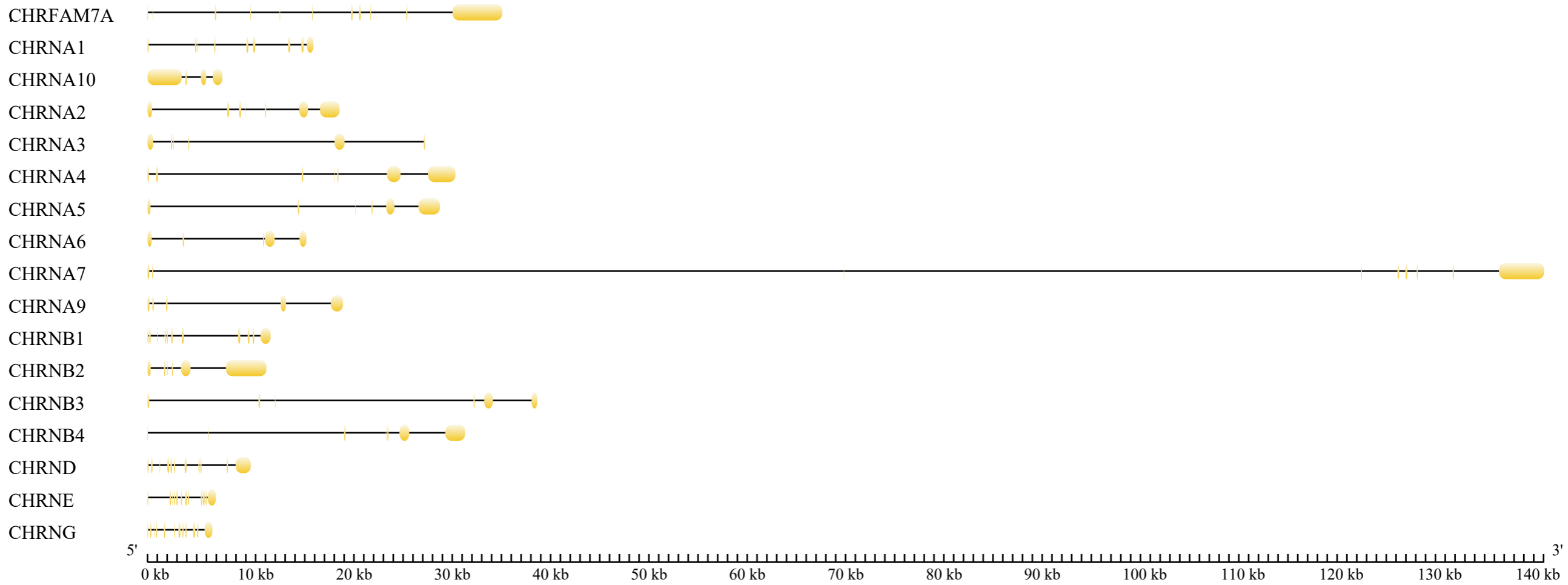

Legend:

Exon

Intron

Supplement: Supplementary file 1 — Additional file 1: Figure S1. Extron-intron structure of nAChR genes from H. sapiens. [file 12864_2019_6278_MOESM1_ESM.pdf]

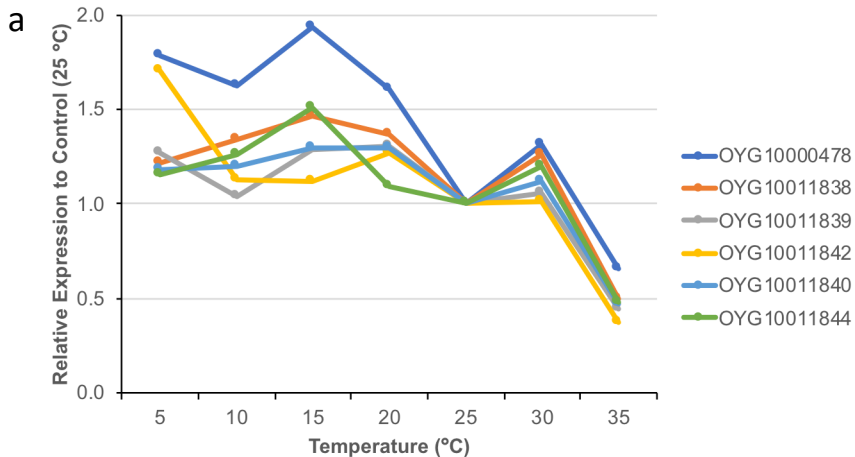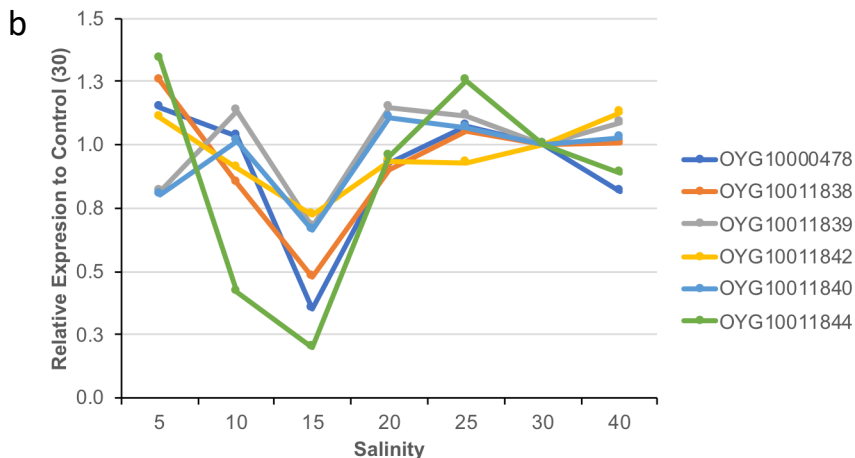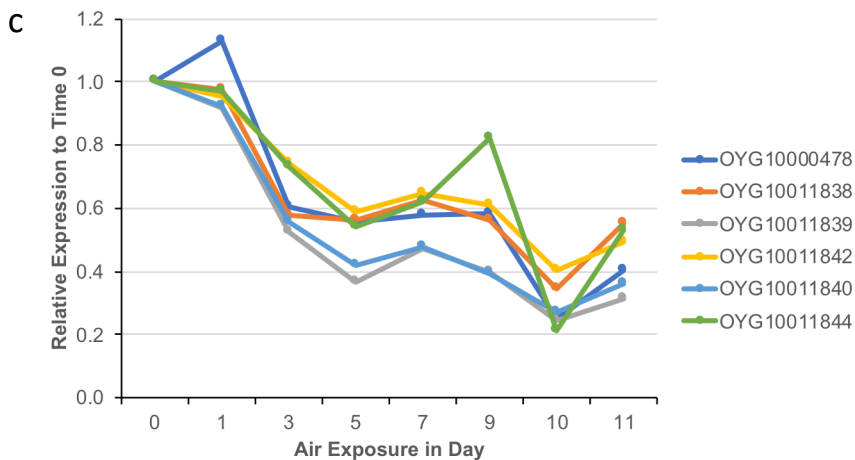

Supplement: Supplementary file 5 — Additional file 5: Figure S5. Expression of six nAChR genes in gills of C. gigas under different environmental conditions (data from Zhang et al. 2012). a Seven days at 5–25 °C or 12 h at 30 and 35 °C; b Seven days under different salinities; and c Air exposure for different durations. [file 12864_2019_6278_MOESM5_ESM.pdf]

**OYG10015788**

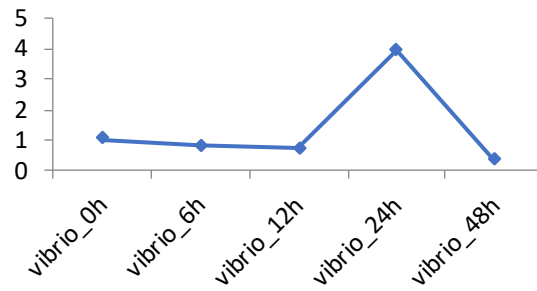

**OYG10015788**

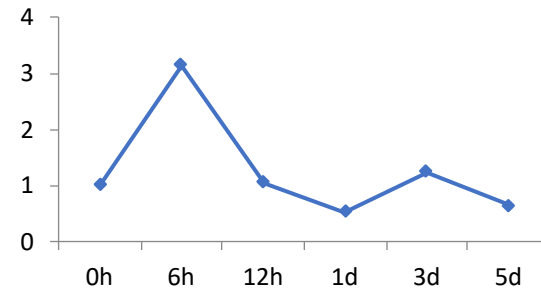

**OYG10015789**

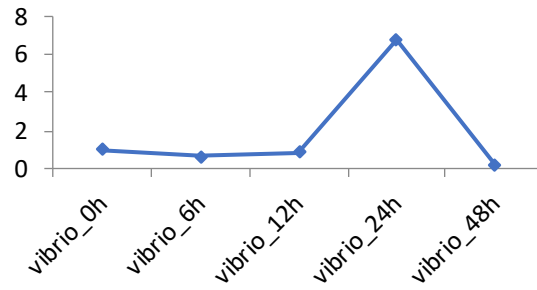

**OYG10018400**

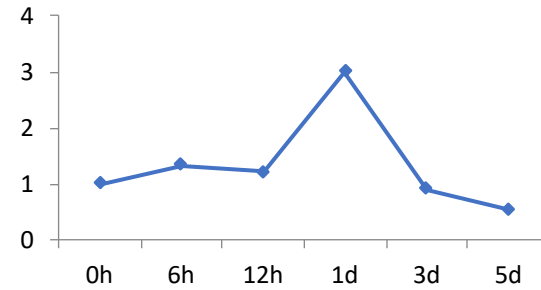

**OYG10023220**

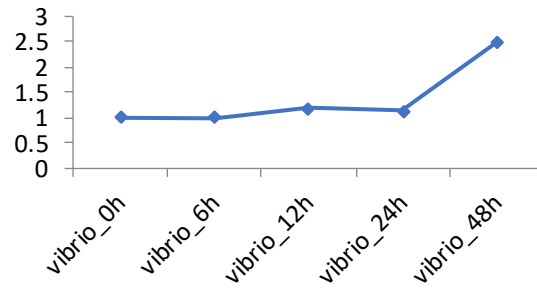

**OYG10023219**

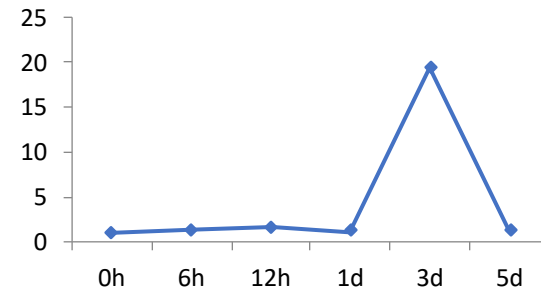

Supplement: Supplementary file 6 — Additional file 6: Figure S6. Expression of nAChR genes in gills of C. gigas in response to infection by pathogens. Left, expression of 3 nAChRs at different times after Vibrio (V. anguillarum, V. tubiashii, V. aestuarianus, V. alginolyticus) challenge (data from Zhang et al. 2015); Right, expression of 3 nAChRs at different times after Ostreid herpesvirus 1-μVar challenge (data from He et al. 2015). Y-axes is expression relative to Time 0. [file 12864_2019_6278_MOESM6_ESM.pdf]
