# Supplementary material for: Massive expansion and diversity of nicotinic acetylcholine receptors in lophotrochozoans
Source: BMC Genomics. 2019 Dec 5;20:937. doi: 10.1186/s12864-019-6278-9 (PMC6896357; doi:10.1186/s12864-019-6278-9)
Supplement: Supplementary file 2 — Additional file 2: Figure S2. Multi-alignment of Cys-loop of nAChR genes from C. gigas and H. sapiens. [file 12864_2019_6278_MOESM2_ESM.pdf]

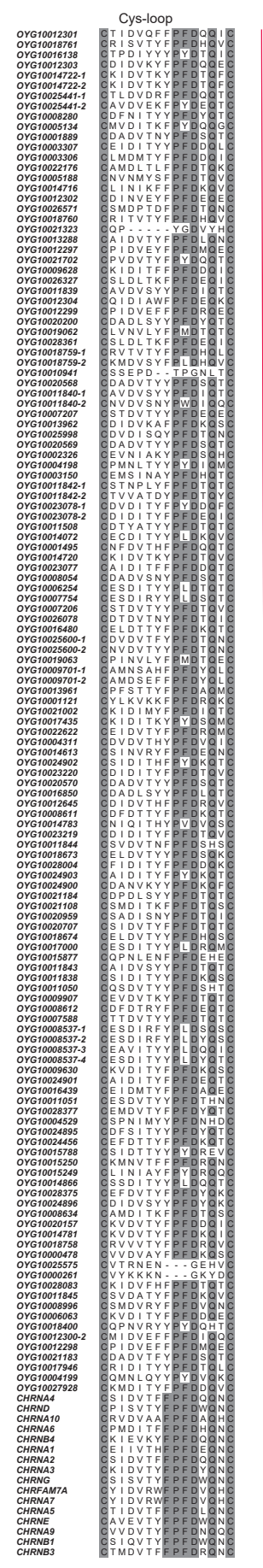
Cys-loop
OYG10012301 CTIDVQFFPFDQQIC
OYG10018761 CRISVTYFPFDHQVC
OYG10016138 CTPDIYYYPYDTQIC
OYG10012303 CDIDVKYFPFDQQEC
OYG10014722-1 CKIDVTKYPFDTQFC
OYG10014722-2 CKIDVTKYPFDTQFC
OYG10025441-1 CTLDVTRFPFDQQTC
OYG10025441-2 CAVDVEKFPYDEQTC
OYG10008280 CDFNITYYPFDYQTC
OYG10005134 CMVDITKFPYDQQGC
OYG10001889 CDADVTNYPFDSQTC
OYG10003307 CEIDITYYPFDDQLC
OYG10003306 CLMDMTYFPFDDQIC
OYG10022176 CAMDLTLFPFDTQKC
OYG10005188 CNVNMYSFPFDTQKC
OYG10014716 CLINIKFFPFDKQVC
OYG10012302 CDINVEYFPFDEQEC
OYG10026571 CSMDPTDFPFDTQNC
OYG10018760 CRITVTYFPFDHQVC
OYG10021323 CQP-----YGDVYHC
OYG10013288 CAIDVTYFPFDLQNC
OYG10012297 CPIDVEYFPFDDQIC
OYG10021702 CPVDVTYFPYDQQTC
OYG10009628 CKIDITFFPFDDQIC
OYG10026327 CSLDLTKFPFDEQIC
OYG10011839 CAVDVSYYPFDIQTC
OYG10012304 CQIDIAWFPFDEQKC
OYG10012299 CPIDVEFFPFDRQEC
OYG10020200 CDADLSYYPFDIQTC
OYG10019062 CLVNVLYFPMDTQTC
OYG10028361 CSLDLTKFPFDEQIC
OYG10018759-1 CRVTVTYFPFDHQLC
OYG10018759-2 CKMDVSYFPLDHQVC
OYG10010941 CSSEPD--TPGNLTC
OYG10020568 CDADVTYYPFDSQTC
OYG10011840-1 CAVDVSYYPFDIQTC
OYG10011840-2 CNVDVSNYPWDIQQC
OYG10007207 CSTDVTYYPFDEQEC
OYG10013962 CDIDVKAFPFDKQSC
OYG10025998 CDVDISQYPFDTQNC
OYG10020569 CDADVTYYPFDSQTC
OYG10002326 CEVNIAKYPFDSQHC
OYG10004198 CPMNLTYYPYDTQIC
OYG10003150 CEMSINAYPFDHQTC
OYG10011842-1 CSTNPLYFPFDTQTC
OYG10011842-2 CTVVATDYPFDTQYC
OYG10023078-1 CDVDITYFPYDDQFC
OYG10023078-2 CDIDITYFPFDEQIC
OYG10011508 CDTYATYYPFDTQTC
OYG10014072 CECDITYFPLDTQTC
OYG10001495 CNFDVTHFPFDQQTC
OYG10014720 CKIDVTKYPFDTQVC
OYG10023077 CAIDITFFPFDDQTC
OYG10008054 CDADVSNYPFDSQTC
OYG10006254 CESDITYYPLDTQTC
OYG10007754 CESDIRYYPLDSQTC
OYG10007206 CSTDVTYYPFDEQEC
OYG10026078 CDTDVTNYPFDTQIC
OYG10016480 CELDTTYFPFDKQTC
OYG10025600-1 CDVDVTFYPFDTQNC
OYG10025600-2 CNVDVTYYPFDTQNC
OYG10019063 CPINVLYFPMDTQEC
OYG10009701-1 CAMNSAHFPFDYQLC
OYG10009701-2 CAMDSEFFPFDYQLC
OYG10013961 CPFSTTYFPFDAQMC
OYG10001121 CYLKVKKFPFDRQKC
OYG10021002 CKIDIMYFPFDIQTC
OYG10017435 CKIDITKYPYDSQMC
OYG10022622 CEIDVTYFPFDRQMC
OYG10004311 CDVDVTHYPFDVQIC
OYG10014613 CSINVRYYPFDTQNC
OYG10024902 CSIDITHFPYDKQTC
OYG10023220 CDIDITYFPFDTQVC
OYG10020570 CDADVTYYPFDSQTC
OYG10016850 CDADLSYYPFDLQTC
OYG10012645 CDIDVTHFPFDRQVC
OYG10008611 CDFDTTYFPFDKQTC
OYG10014783 CNIQITYFPVDTQSC
OYG10023219 CDIDITYFPFDTQVC
OYG10011844 CSVDVTNFPFDSHSC
OYG10018673 CELDVTYYPFDSQKC
OYG10028004 CFIDITYFPFDDQKC
OYG10024903 CAIDITYFPYDKQTC
OYG10024900 CDANVKYYPFDKQFC
OYG10022184 CDPDLSYYPFDTQTC
OYG10021108 CSMDITKFPFDTQSC
OYG10020959 CSADISNYPFDTQIC
OYG10020707 CSIDVTYFPFDTQTC
OYG10018674 CELDVTYYPFDHQSC
OYG10017000 CESDITYYPLDRQMC
OYG10015877 CQPNLENFPFDEHEC
OYG10011843 CAIDVSYYPFDTQTC
OYG10011838 CSIDITYYPFDKQSC
OYG10011050 CQSDVTYYPFDSHTC
OYG10009907 CEVDVTKYPFDTQTC
OYG10008612 CDFDTRYFPFDEQTC
OYG10007588 CTTDVTYYPFDTQTC
OYG10008537-1 CESDIRFYPLDSQSC
OYG10008537-2 CESDIRFYPLDTQSC
OYG10008537-3 CEAVITYYPLDQQIC
OYG10008537-4 CESDITYYPLDYQTC
OYG10009630 CKVDITYFPFDKQSC
OYG10024901 CAIDITYFPFDEQTC
OYG10016439 CEIDMTYFPFDAQEC
OYG10011051 CESDVTYYPFDTHNC
OYG10028377 CEMDVTYYPFDTQTC
OYG10004529 CSPNIMYYPFDNHDC
OYG10024895 CDFSITYYPFDYQTC
OYG10024456 CEFDTTYFPFDKQTC
OYG10015788 CSIDTTYYPYDREVC
OYG10015250 CKMNVTFFPFDRQNC
OYG10015249 CLINIAYFPYDRQQC
OYG10014866 CSSDITYFPLDTQTC
OYG10028375 CEFDVTYFPFDYQKC
OYG10024896 CDIDVSYYPFDYQKC
OYG10008634 CAMDITKFPFDTQSC
OYG10020157 CKVDVTYFPFDDQIC
OYG10014781 CKVDVTYFPFDKQIC
OYG10018758 CRVVVTYFPFDRQSC
OYG10000478 CVVDVAYFPFDSQSC
OYG10025575 CVTRNEN---GEHVC
OYG10000261 CVYKKKN---GKYDC
OYG10028083 CKIDVFHFPFDTQTC
OYG10011845 CSVDATYFPFDKQVC
OYG10008996 CSMDVRYFPFDVQNC
OYG10006063 CKVDITYFPFDDQEC
OYG10018400 CPVNVRYFPYDTQNC
OYG10012300-2 CMIDVEFFPFDIQQC
OYG10012298 CPIDVEFFPFDMQEC
OYG10021183 CDADVTFYPFDSQTC
OYG10017946 CRIDITYYPFDTQLC
OYG10004199 CQMNLQYYPYDVQKC
OYG10027928 CKMDITYFPFDDQVC
CHRNA4 CSIDVTYFPFDWQNC
CHRND CPISVTYFPFDWQNC
CHRNA10 CRVDVAAFPFDAQHC
CHRNA6 CPMDITFFPFDHQNC
CHRNB4 CKIEVKYFPFDQQNC
CHRNA1 CEIIVTHFPFDEQNC
CHRNA2 CSIDVTFFPFDQQNC
CHRNA3 CSIDVTYFPFDWQNC
CHRNG CSISVTYFPFDWQNC
CHRFAM7A CYIDVRWFPFDVQHC
CHRNA7 CYIDVRWFPFDVQHC
CHRNA5 CTIDVTFFPFDLQNC
CHRNE CAVEVTYFPFDWQNC
CHRNA9 CVVDVTYFPFDNQQC
CHRNB1 CSIDVTYFPFDWQNC
CHRNB3 CTMDVTFFPFDRQNC
Oyster
Human
