# Supplementary material for: Massive expansion and diversity of nicotinic acetylcholine receptors in lophotrochozoans
Source: BMC Genomics. 2019 Dec 5;20:937. doi: 10.1186/s12864-019-6278-9 (PMC6896357; doi:10.1186/s12864-019-6278-9)
Supplement: Supplementary file 3 — Additional file 3: Figure S3. Multi-alignment of alpha nAChR genes from H. sapiens. [file 12864_2019_6278_MOESM3_ESM.pdf]

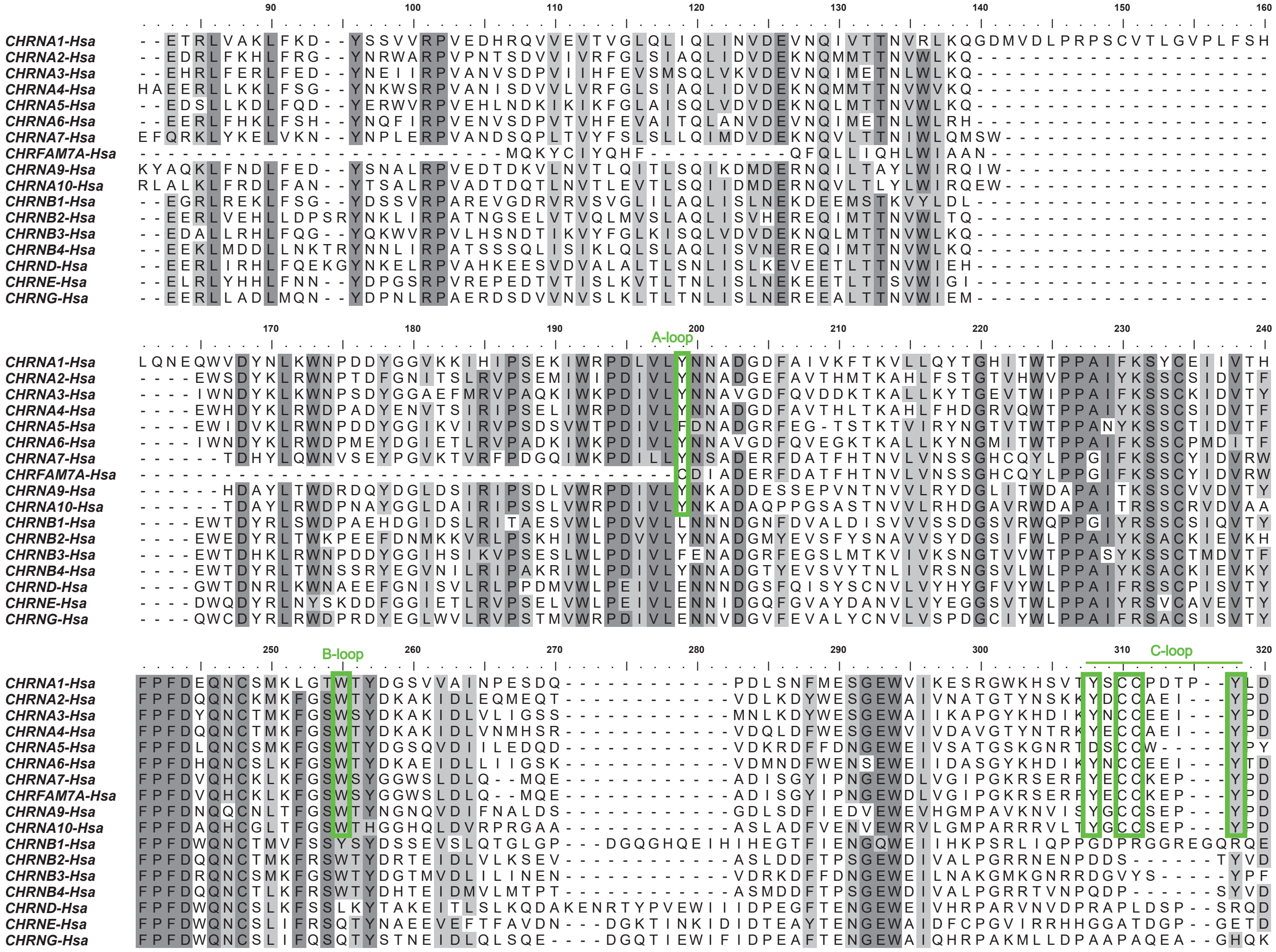

90
100
110
120
130
140
150
160
170
180
190
200
210
220
230
240
250
260
270
280
290
300
310
320
A-loop
B-loop
C-loop
CHRNA1-Hsa
CHRNA2-Hsa
CHRNA3-Hsa
CHRNA4-Hsa
CHRNA5-Hsa
CHRNA6-Hsa
CHRNA7-Hsa
CHRFAM7A-Hsa
CHRNA9-Hsa
CHRNA10-Hsa
CHRNB1-Hsa
CHRNB2-Hsa
CHRNB3-Hsa
CHRNB4-Hsa
CHRND-Hsa
CHRNE-Hsa
CHRNG-Hsa
