# Supplementary material for: Massive expansion and diversity of nicotinic acetylcholine receptors in lophotrochozoans
Source: BMC Genomics. 2019 Dec 5;20:937. doi: 10.1186/s12864-019-6278-9 (PMC6896357; doi:10.1186/s12864-019-6278-9)
Supplement: Supplementary file 4 — Additional file 4: Figure S4. Sequence diversity at and around ACh binding sites in 33 nAChR genes of C. gigas with the sites conserved. Sequences marked by the red underline is the Cys-loop. Amino acids in green boxes are ACh binding sites. Hsa, H. sapiens; Tma, Torpedo marmorata. Genes in purple are nAChRs with completely conserved principal binding sites. [file 12864_2019_6278_MOESM4_ESM.pdf]

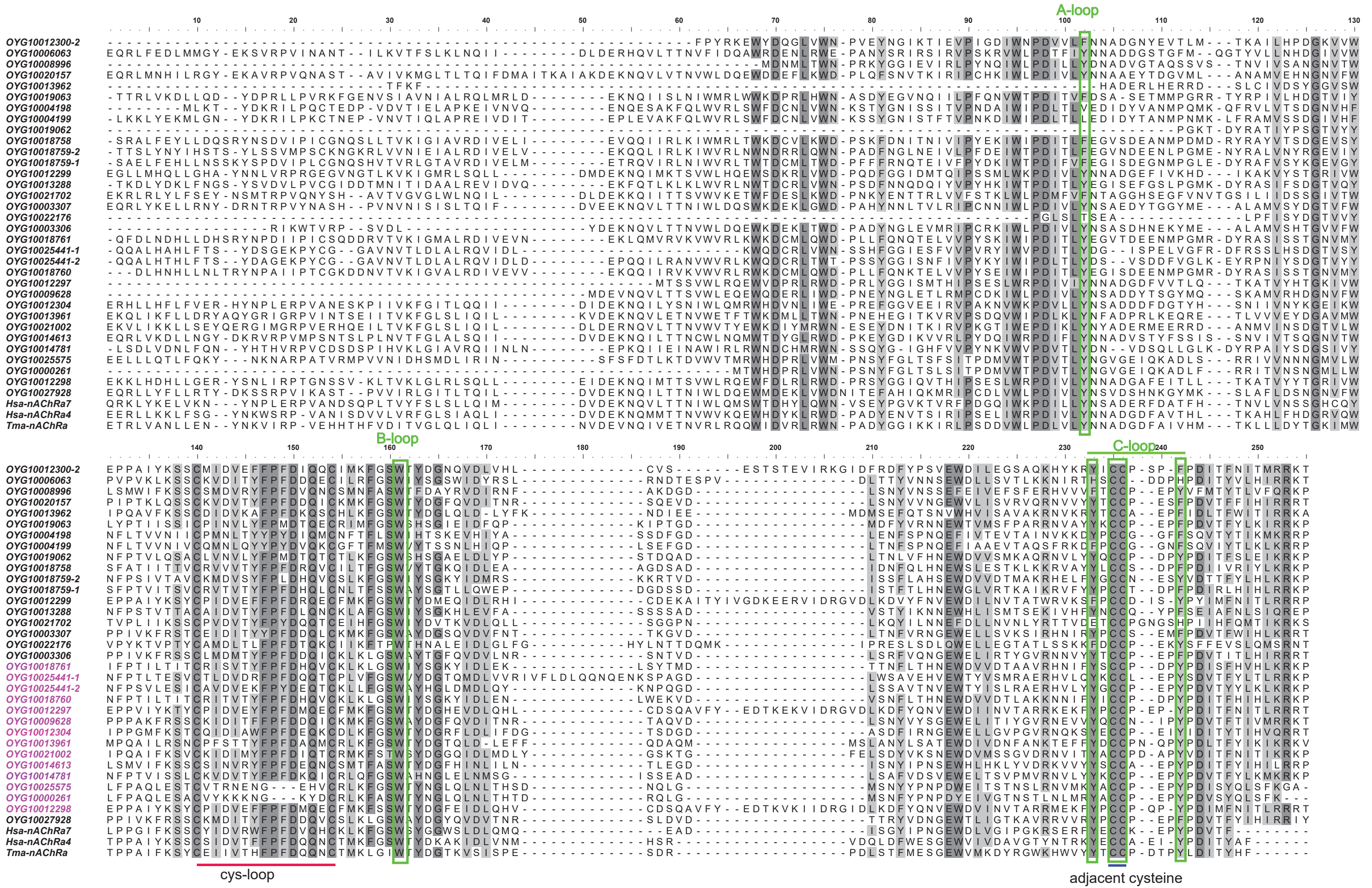
A-loop
B-loop
C-loop
cys-loop
adjacent cysteine
